# Vitamin D status in pediatric irritable bowel syndrome

**DOI:** 10.1371/journal.pone.0172183

**Published:** 2017-02-13

**Authors:** Benjamin Udoka Nwosu, Louise Maranda, Ninfa Candela

**Affiliations:** 1 Division of Endocrinology, Department of Pediatrics, University of Massachusetts Medical School, Worcester, Massachusetts, United States of America; 2 Department of Quantitative Health Sciences, University of Massachusetts Medical School, Worcester, Massachusetts, United States of America; 3 Division of Gastroenterology, Department of Pediatrics, University of Massachusetts Medical School, Worcester, Massachusetts, United States of America; University of Alabama at Birmingham, UNITED STATES

## Abstract

**Importance:**

Irritable bowel syndrome (IBS) is associated with significant morbidity in children and adolescents, and the therapeutic efficacy of available treatment options is limited. The role of vitamin D supplementation in pediatric IBS is unclear as the vitamin D status of pediatric patients with IBS is unknown. Equally, the relationship of vitamin D status with psychosomatic symptoms in children and adolescents is unclear.

**Aim:**

To characterize the vitamin D status of pediatric patients with IBS using a case-control study design.

**Hypothesis:**

Serum 25-hydroxyvitamin D [25(OH)D] concentration will be similar between patients with IBS and controls.

**Subjects and methods:**

A retrospective case-controlled study of 116 controls (age 14.6 ± 4.3 y), female (n = 67; 58%) and 55 subjects with IBS (age 16.5 ± 3.1y), female (n = 44; 80%). Overweight was defined as BMI of ≥85^th^ but <95^th^ percentile, and obesity as BMI ≥95^th^ percentile. Vitamin D deficiency was defined as 25(OH)D of <50 nmol/L, while seasons of vitamin D draw were categorized as summer, winter, spring, and fall. Major psychosomatic manifestations included in the analysis were depression, anxiety, and migraine.

**Results:**

More than 50% of IBS subjects had vitamin D deficiency at a cut-off point of <50 nmol/L (53% vs. 27%, p = 0.001); and >90% of IBS subjects had vitamin D deficiency at a cut-off point of <75 nmol/L (93% vs. 75%, p = 0.006). IBS subjects had significantly lower mean 25(OH)D: 53.2 ± 15.8 nmol/L vs. 65.2 ± 28.0 nmol/L, p = 0.003; and albumin: 6.2 ± 0.6 vs. 6.5 ± 0.6 μmol/L, p = 0.0.01. IBS subjects with migraine had significantly lower mean 25(OH)D concentration compared to controls (p = 0.01). BMI z-score was similar between the controls and IBS subjects (0.5 ± 1.4 vs. 1.2 ± 2.9, p = 0.11).

**Conclusions:**

Pediatric patients with IBS had significantly lower 25(OH)D concentration compared to controls despite having similar mean BMI values as controls. Only 7% of the children and adolescents with IBS were vitamin D sufficient, and >50% of the subjects with IBS had vitamin D deficiency. This is a much higher prevalence of vitamin D deficiency compared to IBD and other malabsorption syndromes. Monitoring for vitamin D deficiency should be part of the routine care for patients with IBS. Randomized control trials are warranted to determine the role of adjunctive vitamin D therapy in pediatric IBS.

## Introduction

The vitamin D status of children and adolescents with irritable bowel disease (IBS) is not known, and the relationship of vitamin D status with associated psychosomatic symptomatology in IBS is unclear. Irritable bowel syndrome (IBS) is a non-inflammatory, functional disorder of the gastrointestinal tract that affects 10–15% of people in the industrialized world[1]. The pathogenesis of IBS remains an enigma and the mechanism responsible for the flares and associated psychosomatic manifestations such as depression, anxiety, and migraines, are poorly understood[2]. The etiopathogenesis of IBS is multifactorial and is believed to involve the dysfunction of the brain-gut axis, enteric neuromuscular system, nonspecific immune activation, and altered intraluminal environment[2].

Adult subjects with IBS have a high prevalence of vitamin D deficiency[3] and vitamin D supplementation is reported to be associated with improvements in various quality of life indices in these patients[2, 4]. This is crucial as the therapeutic efficacy of drugs used to manage IBS is limited and the response to these agents vary between patients[5].

Regrettably, the vitamin D status of children and adolescents with IBS has not been characterized, and the non-dietary determinants of vitamin D in this population are not fully described. Equally, the relationship between vitamin D status and psychosomatic symptomatology in pediatric patients with IBS is not clear.

Additionally, there has not been a rigorous comparison of vitamin D status in children and adolescents with either inflammatory bowel disease (IBD) or irritable bowel syndrome (IBS), as these two disease states are easily confused with each other. This is important as our group has previously characterized the vitamin D status in IBD and found no significant difference in mean serum 25(OH)D concentration between children and adolescents with IBD and controls[6]. However, we noted that IBD subjects with elevated erythrocyte sedimentation rate (ESR) had significantly lower 25(OH)D concentration than controls which led to the recommendation that IBD subjects with elevated ESR should be monitored for vitamin D deficiency[6].

To address the above knowledge gap and help clear the confusion on vitamin D status in IBD and IBS, we designed this study to determine the vitamin D status of pediatric patients with IBS only; characterize the determinants of vitamin D status in this condition, and investigate the relationship between vitamin D status and psychosomatic manifestations in IBS. We hypothesized that vitamin D status would be similar in pediatric patients with IBS and controls.

## Subjects and methods

### Ethics statement

This study protocol was approved by the Institutional Review Board of the University of Massachusetts which granted the approval for the retrospective review of records from patients’ case records. Subjects’ data were anonymized and de-identified before analysis.

### Subjects

This study involved the extraction and review of medical records of pediatric patients of ages 6–21 years with a confirmed diagnosis of IBS between January 1, 2007 and June 30, 2013 at the Children’s Medical Center Database of the UMassMemorial Medical Center, Worcester, Massachusetts, USA.

Study subjects (n = 55; 11 males) were included if they had a confirmed diagnosis of IBS. The diagnosis of IBS was established using the Rome 3 Criteria as follows: recurrent abdominal pain or discomfort for at least 3 days per month in the preceding 3 months, and associated with two or more of the following: (a) symptomatic improvement with defecation, (b) onset associated with a change in frequency of stool, and (c) onset associated with a change in the form or appearance of stool[7, 8]. Medical records were further reviewed for confirmed diagnosis of classic comorbidities of IBS: anxiety disorder, depression, and migraine headache. Laboratory analytes including serum 25(OH)D, alanine transaminase (ALT), albumin, and erythrocyte sedimentation rate (ESR) were available for all subjects, and were drawn on the same day during routine clinic visit.

As described in detail previously[6, 9], the control group (n = 116; 67 females) consisted of a cohort of healthy children and adolescents attending the well-child clinic of the Children’s Medical Center. The controls were selected randomly through a systematic sampling scheme, that involved the alphabetization of the list of the control subjects, and then selecting every 5^th^ individual for inclusion in the study. None of the control subjects had a diagnosis of IBS, IBD, or celiac disease. The controls were neither receiving vitamin D supplementation nor steroid therapy at the time of their inclusion in the study. The control subjects had serum 25(OH)D, ALT, and albumin drawn as part of routine comprehensive metabolic panel and vitamin D estimation during a clinic visit between January 1, 2007 and June 30, 2013.

The exclusion criteria for this study included pregnant or lactating subjects, patients with chronic liver disease, malabsorption syndrome, chronic liver disease, IBD, diseases affecting calcium and/or vitamin D metabolism, and any treatment with steroids, vitamin D or calcium supplementation prior to the date of 25(OH)D measurement. Of the 65 subjects with IBS who met the inclusion criteria, 10 subjects were excluded based on the aforementioned exclusion criteria. Fifty-five subjects were included in the analysis.

The date of 25(OH)D measurement was used to determine the age of study subjects; and the duration of disease was designated as the interval between the diagnosis of IBS and the date of first 25(OH)D estimation. Seasonal variations in serum 25(OH)D concentration was taken into consideration by categorizing subjects’ date of vitamin D drawn according to the season as follows: summer (June 22-September 21), fall (September 22 –December 21), winter (December 22- March 21), and spring (March 22 –June 21)[10].

### Anthropometry

As described in detail previously[6, 9], weight was measured to the nearest 0.1 kg using an upright scale. Height was measured to the nearest 0.1 cm using a wall-mounted stadiometer that was calibrated daily. BMI was calculated from the formula: weight/height^2^ (kg/m^2^), and expressed as standard deviation score (SDS) for age and sex, based on National Center for Health Statistics (NCHS) data.[11] Overweight was defined as BMI of ≥85^th^ but <95^th^ percentile, and obesity was defined as BMI of ≥95^th^ percentile for age and gender.

### Assays

Assay protocol has been previously described[6, 9]. Briefly, serum 25(OH)D concentration was analyzed using 25-hydroxy chemiluminescent immunoassay (DiaSorin Liaison; Stillwater, Minnesota), which measures total serum 25(OH)D content as it detects both metabolites of 25(OH)D: 25(OH)D_2_ and 25(OH)D_3_. It has an intra- and inter-assay coefficients of variation of 5% and 8.2% respectively, and a functional sensitivity of 10 nmol/L. The characterization of vitamin D status for this study was based on The Endocrine Society Clinical Practice Guideline which defined vitamin D status using serum 25(OH)D values as follows: vitamin D deficiency < 20 ng/mL (50 nmol/L), insufficiency 20–29.9 ng/mL (50–74.5 nmol/L), and sufficiency ≥ 30 ng/mL (75 nmol/L)[12]. This characterization is similar to the classification of vitamin D status by the Institutes of Medicine and the American Academy of Pediatrics which denote vitamin D deficiency as 25(OH)D <50 nmol/L; or sufficiency, 25(OH)D >50 nmol/L[13, 14].

ESR analyzer, Vesmatic 20 (Clinical Data, Inc., Smithfield, RI) was used to quantify ESR according to standard protocol. Beckman Coulter AU System ALT procedure (Beckman Coulter, Inc., 250 S. Kraemer Blvd. Brea, CA 92821, USA) was used to measure ALT, while albumin concentrations were measured in a Roche/Hitachi 917 chemistry analyzer (Roche Diagnostic Corporation, Indianapolis, IN) according to standard protocol.

### Statistical analyses

Means and standard deviations (SD) were calculated for descriptive summary statistics and 25(OH)D measurements. Univariate and multivariate comparisons on anthropometrics, 25(OH)D, and other variables were conducted using ANOVA and two-tailed student’s t-test respectively. Height, weight, and BMI data were expressed as z-scores. Race, gender proportionality, and seasons of blood draw were compared using Pearson Chi-Square. Simple and multiple linear regression models, describing the relationships between anthropometric and biochemical parameters, were specified. Data were expressed as mean ± SD. All statistical analyses were performed using the SPSS Predictive Analytics SoftWare v.23 (IBM Corporation, Armonk, NY).

## Results

### Comparison of the characteristics of subjects with IBS vs. controls

There were no differences in height SDS, weight SDS, and BMI SDS between the controls and IBS subjects (Table 1). There were equally no differences in racial composition of subjects and controls, and the season of 25(OH)D measurement. Subjects with IBS were older (p = 0.004), had lower serum concentration of albumin (p = 0.01), and a significantly higher prevalence of vitamin D deficiency at a cutoff point of ≤50 nmol/L (53% vs. 27%, p = 0.001). Only 7% of IBS patients met the criterion for vitamin D sufficiency, 25(OH)D of ≥75 nmol/L, compared to 25% of controls (p = 0.006). The control group consisted of 75% Non-Hispanic Whites (NHW), while the IBS cohort consisted of 78% NHW (p = 0.65).

**Table 1:** A comparison of the characteristics of subjects with irritable bowel syndrome and controls.

Parameter	IBS (n = 55)	Controls (n = 116)	*p* value
Age (years)	17 ± 3	15 ± 4	**0.004**
Height SDS	-0.2 ± 1	0.0 ± 1	0.39
Weight SDS	0.7 ± 1	0.4 ± 2	0.27
BMI SDS	1 ± 3	0.5 ± 1	0.11
Sex: (% Males)	20	42	**0.004**
Race (% White)	78	75	0.65
ALB (μmol/L)	6 ± 1	7 ± 1	**0.01**
ALT (U/L)	19 ± 20	19 ± 33	0.98
25(OH)D (nmol/L)	53 ± 16	65 ± 28	**0.003**
25(OH)D ≤50 nmol/L (%)	53	27	**0.001**
25(OH)D ≤75 nmol/L (%)	93	75	**0.006**
Seasons: (Summer-Fall, %)	47	56	0.28

IBS irritable bowel syndrome; SDS standard deviation score; 25(OH)D 25-hydroxyvitamin D; ALT alanine transaminase; ALB albumin, significant ***p*** values are bolded.

### The effect of BMI on serum 25(OH)D concentration in IBS vs. controls

**Fig 1:**
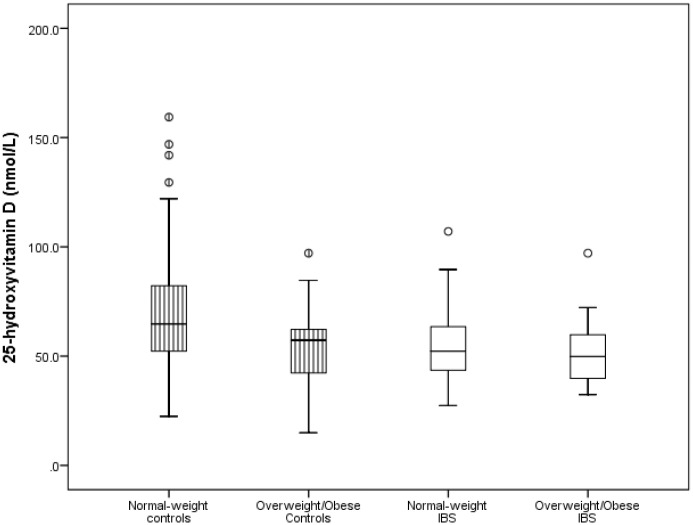
Box plot of the comparison of 25-hydroxyvitamin D values of patients with irritable bowel syndrome (IBS) and controls stratified by body mass index (BMI). This figure shows that serum 25(OH)D concentration was significantly higher in the normal-weight controls compared to the overweight/obese controls (69.8 ± 27.5 nmol/L vs. 58.3 ± 27.6, p = 0.03), and to both the normal-weight and overweight/obese IBS subjects. Serum 25(OH)D concentration was similar between the normal-weight IBS- and overweight/obese IBS subjects (p = 0.91) Note: 50 nmol/L = 20 ng/mL. Circles represent standard deviations.

When subjects were stratified into normal-weight (BMI <85^th^ percentile) and overweight/obese (BMI >85^th^ percentile), serum 25(OH)D concentration was significantly higher in the normal-weight controls compared to the overweight/obese controls (69.8 ± 27.5 nmol/L vs. 58.3 ± 27.6, p = 0.03), and to both the normal-weight and overweight/obese IBS subjects. Serum 25(OH)D concentration was similar between the normal-weight IBS- and overweight/obese IBS subjects (p = 0.91) (Fig 1). The normal-weight IBS subjects had lower 25(OH)D concentration compared to the normal-weight controls (55.4 ± 17.5 vs. 69.8 ± 27.5 nmol/L, p = 0.05), while normal-weight controls had significant higher 25(OH)D concentration compared to the overweight/obese IBS patients (69.8 ± 27.5 nmol/L vs. 51.1 ± 14.0, p = 0.004).

### The relationship between psychosomatic symptoms and 25(OH)D concentration in IBS

**Fig 2:**
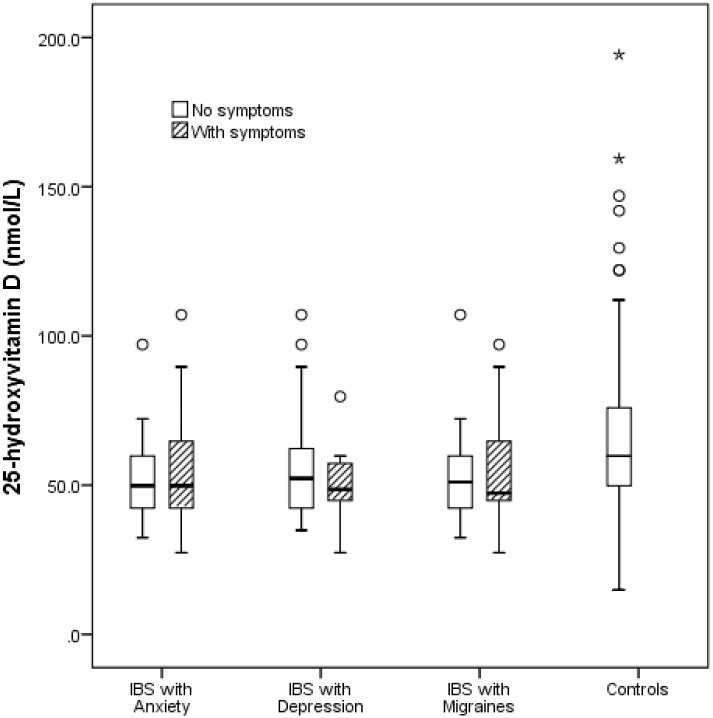
Boxplot of the comparison of 25(OH)D concentrations between the controls and IBS subjects with psychosomatic manifestations. This figure shows a significant difference in serum 25(OH)D between the 4 groups: IBS subjects with anxiety (n = 17), IBS subjects with depression (n = 14), and IBS subjects with migraines (n = 13), and the controls, (ANOVA, p = 0.004). *Post hoc* analysis revealed a significant difference in 25(OH)D between the controls and subjects with IBS and migraines (p = 0.01), but not for IBS patients with depression (p = 0.08), or IBS patients with anxiety (p = 0.44). Hatched boxes represent subjects with symptoms, while clear boxes represent subjects with no symptoms. Circles and asterisks represent standard deviations.

Fig 2 shows the comparison of the 25(OH)D concentrations between the controls, IBS subjects with anxiety (n = 17), IBS subjects with depression (n = 14), and IBS subjects with migraines (n = 13). There was a significant difference in serum 25(OH)D between the 4 groups (ANOVA, p = 0.004). *Post hoc* analysis revealed a significant difference in 25(OH)D between the controls and subjects with IBS and migraines (p = 0.01), but not for IBS patients with depression (p = 0.08), or IBS patients with anxiety (p = 0.44).

### Regression analysis of 25(OH)D vs. ESR, albumin, and alanine transaminase

A regression analysis of the IBS cohort showed no significant relationship between 25(OH)D and ESR (R^2^ = 0.03, β = -0.05, p = 0.31), ALT (R^2^ = 0.043, β = 0.006, p = 0.94) and albumin (R^2^ = 0.046, β = 0.057, p = 0.92) after adjusting for age, gender, race, season, and BMI SDS.

### Seasons and 25(OH)D concentration in controls and IBD subjects

**Fig 3:**
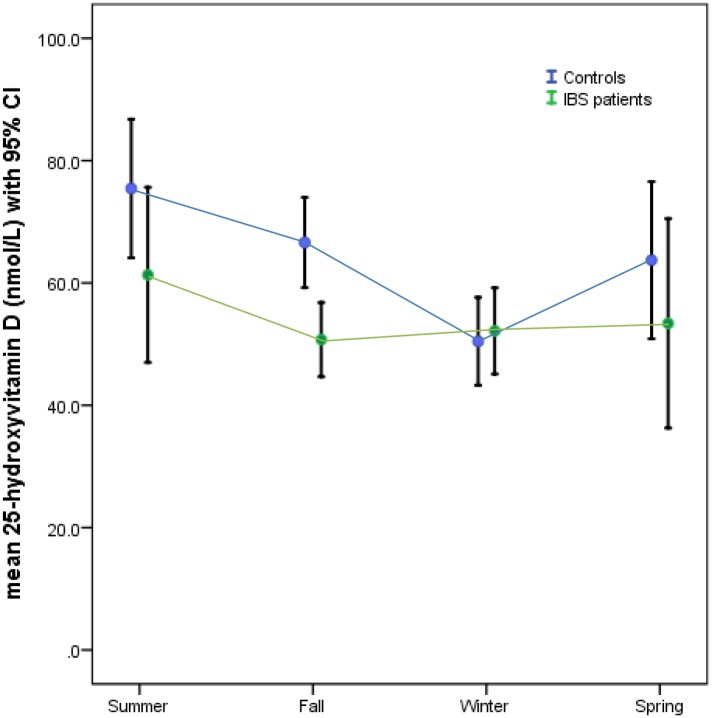
Graph of the relationship between the seasons and serum 25(OH)D concentration in patients with IBS and controls. Serum 25(OH)D was significantly lower in the subjects with IBS compared to controls (ANOVA, p = 0.04). Both groups had higher serum concentration of 25(OH)D in the summer compared to winter (ANOVA, p = 0.04). There were no interactions between the groups and seasons (ANOVA, p = 0.24).

Fig 3 shows the relationship between seasons and serum 25(OH)D concentration in patients with IBS and controls. There were significant differences in 25(OH)D concentrations between the seasons (ANOVA, p = 0.04), with higher 25(OH)D concentration noted in the summer than in winter in both the controls and IBS subjects. Serum 25(OH)D was significantly lower in the subjects with IBS compared to controls (ANOVA, p = 0.04). There were no interactions between the groups and seasons (ANOVA, p = 0.24).

### Patient’s sex and 25(OH)D concentration

Analysis of the differences in 25(OH)D by sex between the controls and the IBS subjects showed no difference in 25(OH)D between the male and female groups (ANOVA, p = 0.42). Female controls, however, had a significantly higher 25(OH)D than female IBS subjects (p = 0.002) while 25(OH)D was similar between the male controls and male IBS subjects (p = 0.18). There was no difference between serum 25(OH)D concentration between the male and female patients with IBS: 52.3 ± 7.2 nmol/L vs. 53.5 ± 7.3 nmol/L, (p = 0.85).

### Race/Ethnicity and 25(OH)D concentration

When subjects were divided into non-Hispanic whites (NHW) and non-whites, the NHW controls (n = 87) had significantly higher 25(OH)D concentration than NHW IBS subjects (n = 43) (68.7 ± 24.8 vs. 54.7 ± 15.8 nmol/L, p <0.001), while 25(OH)D concentration was similar between the non-white controls (n = 29) vs. non-white IBS subjects (n = 12) (55.3 ± 34.3 vs. 47.9 ± 15.2, p = 0.35).

### The response of IBS subjects to vitamin D supplementation

**Fig 4:**
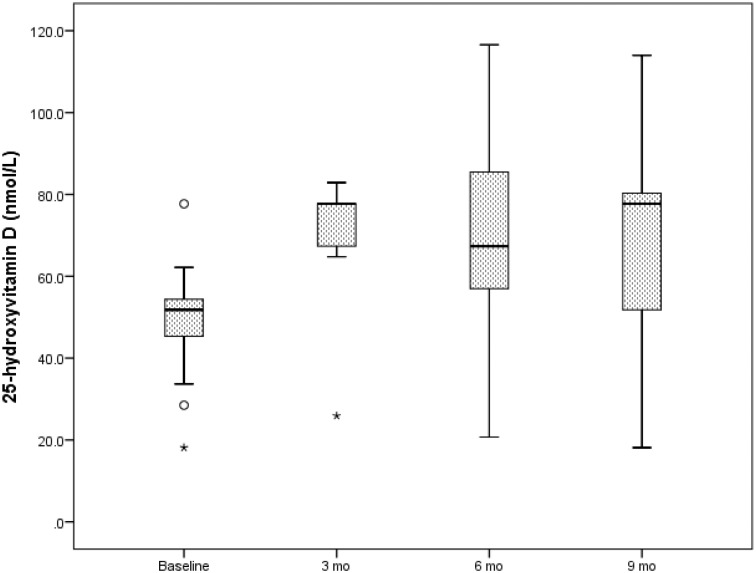
Box plot of the response of subjects with IBS to vitamin D supplementation. There was a significant rise in 25(OH)D from baseline to 3 months, 45.4 ± 14.4 nmol/L to 68.8 ± 14.4 nmol/L, p = 0.002. This increase was sustained through the 9-month observation, consistent with adequate absorption of ingested vitamin D.

Fig 4 shows the change in serum 25(OH)D concentration in 13 subjects with IBS who received vitamin D supplementation using either ergocalciferol or cholecalciferol of doses of 1000 IU to 4000 IU per day, and were monitored over a 9-month period. In the first 3 months of treatment, there was a significant increase in 25(OH)D concentration from 45.4 ± 14.4 nmol/L to 68.8 ± 14.4 nmol/L, (p = 0.002). This response was maintained through the 9-month duration of monitoring.

## Discussion

This is the first study to characterize the vitamin D status of children and adolescents with IBS.

The primary finding from this study is that 1 out of every 2 pediatric patients with IBS has vitamin D deficiency compared to 1 out of every 4 healthy children and adolescents without IBS. Additionally, only 7% of subjects with IBS had vitamin D sufficiency compared to 25% of controls. This is particularly striking as BMI SDS was similar for both the IBS subjects and controls, thus excluding the diminutive effect of BMI on 25(OH)D concentration as the reason for the lower vitamin D concentration in IBS. The lack of a diminutive effect of increasing BMI on serum 25(OH)D in patients with IBS is interesting as this phenomenon has been described only in children and adolescents with inflammatory bowel disease[6], but not in pediatric patients with lactose intolerance[15] or in healthy subjects[15, 16].

The etiology of vitamin D deficiency in pediatric patients with IBS is not clear. It is likely that the vitamin D deficiency described in our cohort resulted from a combination of limited intake of vitamin D because of restricted dietary options and lifestyle habits that limit subjects’ exposure to sunlight[17–20]. A previous study described limited dietary food choices in patients with IBS[17], with special emphasis on limited dairy intake[21], as well as deficiencies in micronutrients such as iron, vitamin B12, and zinc[22]. This is in agreement with a recent study showing significantly lower serum 25(OH)D concentration in vegans compared to meat eaters[23].

Because IBS is functional disorder, the underlying mechanism for vitamin D deficiency in this disorder is not known. Some of the postulated causes of vitamin D deficiency in gastrointestinal disorders include intestinal inflammation, dark skin pigmentation, insufficient solar UV-B exposure, winter season, hypoalbuminemia, and secondary hyperparathyroidism which accelerated the conversion of 25(OH)D to 1,25-dihydroxyvitamin D, thereby exhausting the vitamin D stores[24]. The significantly lower serum albumin concentration in our IBS cohort suggests that hypoalbuminemia may play a role in the pathogenesis of vitamin D deficiency in IBS in children and adolescents. Vitamin D is transported in serum as vitamin D binding protein and thus, low serum albumin concentration, could diminish vitamin D concentration. Serum albumin is a marker of both protein-losing enteropathy and liver function, and has been proposed, along with ESR[6], as a primary determinant of vitamin D status in inflammatory bowel disease[25, 26]. Recent advances in vitamin D research have reported novel pathways of vitamin D metabolism[27–29] that challenge the classical pathway of vitamin D metabolism from the photolysis of 7-dehydrocholesterol to form vitamin D_3_ and then 25(OH)D and 1,25-dihydroxyvitamin D. These non-classical pathways involve the generation of intermediate precursors such as CYP11A1-derived secosteroids, and the initiation of novel pathways of vitamin D_3_ metabolism by P450 side-chain cleavage enzyme and subsequent modification of the precursors by CYP27B1 enzyme system. It is unknown whether these pathways are impaired in patients with IBS.

This study suggests that the non-dietary determinants of vitamin D in pediatric IBS include hypoalbuminemia, dark skin pigmentation, winter seasons, and possibly the female sex. The role of the female sex is important as 80% of the patients with IBS in our study is of the female sex. Serum 25(OH)D was significantly higher in female controls compared to female IBS subjects, but was similar between male controls and male IBS subjects. The racial disparities in 25(OH)D concentration between the controls and subjects with IBS is related to differences in UVB production of vitamin D[30].

Another novel finding from this study is that subjects with IBS and migraine have significantly lower vitamin D level compared to the controls. Though the etiopathogenetic basis for the co-occurrence of psychiatric and somatic symptoms in IBS is unknown[31], the high frequency of the occurrence[32] of these disorders in IBS suggests a common etiological basis that could be addressed with a unified therapeutic approach[2, 33]. Given that 80% of the IBS cohort is of female sex, and that migraine occurs at a higher rate in females[34], it is possible that migraine may occur at a higher frequency in female subjects with IBS and vitamin D deficiency[35]. Serum 25(OH)D concentration was similar between the male and female subjects with IBS in this study. More studies are needed to determine whether adjunctive vitamin D therapy could improve the overall wellbeing of pediatric patients with IBS.

This study found no significant relationship between 25(OH)D and ALT, ESR, and albumin after adjusting for confounders. This is an important area of comparison between IBS and IBD. We have previously reported a significant inverse relationship between 25(OH)D and ESR, but not for ALT and albumin, in children and adolescents with IBD[36]. It is possible that the lack of a relationship between 25(OH)D and ESR in IBS is due to the functional nature of IBS, in contrast to IBD which is primarily marked by intestinal inflammation[36]. The role of inflammation on vitamin D status in IBD was noted in our previous study which showed a significantly lower 25(OH)D concentration in IBD patients with elevated ESR compared to controls[6]. The lack of a significant relationship between 25(OH)D and albumin in IBS is similar to the findings of a study on albumin and 25(OH)D in inflammatory bowel disease [37]; and the absence of a significant relationship between ALT and 25(OH)D in IBS is also consistent with a report that characterized the relationship between vitamin D and liver enzymes in adult patients with liver disease[38]. The seasonal variations in 25(OH)D in IBS subjects is similar to the pattern described in published reports [36, 39, 40]. Equally, the significantly elevated serum 25(OH)D concentration in NHW subjects is consistent with reports from previous studies [41, 42].

This study has a number of limitations. First, the retrospective, cross-sectional design for baseline comparisons limits causal inference on the effects of the studied parameters on vitamin D status. However, the longitudinal analysis of vitamin D status following vitamin D supplementation over 9 months suggests that vitamin D supplementation can improve and maintain normal vitamin D status in children and adolescents with IBS. In this study, subjects with IBS received either ergocalciferol or cholecalciferol. Recent studies suggest that the use of cholecalciferol is more efficacious in raising and maintaining serum 25(OH)D concentration[43–45]. The approach to the management of vitamin D deficiency in IBS is similar to the established guidelines for the general population[12]. The second shortcoming of this study is that food-recall questionnaires were not administered to the children to accurately determine each subject’s dietary vitamin D intake. This is particularly important as vitamin D concentration may be diminished in patients with severe dietary restrictions, as well as in those with lifestyle choices that limit sun exposure. Equally, there were no data on the consumption of micronutrients as these may also be deficient in patients with IBS. Such data could have been used to correlate the degree of vitamin D deficiency state to concomitant micronutrient deficiency. Third, data on parathyroid hormone levels were not available as these could be elevated in states of vitamin D deficiency. Fourth, this study was based on a small sample size and this limits its generalizability. The IBS cohort was older than the controls, however, all relevant analyses were adjusted for age and other covariates. Published studies showed that age has no influence on vitamin D status[26]. The results of this study were not adjusted for the type of medical therapy, or pubertal status, as earlier studies found no relationships between 25(OH)D and these parameters in children and adolescents with gastrointestinal disorders [25, 39, 46].

The strengths of this study include its case-control design that allowed for the comparison of key parameters in patients with IBS to similar parameters in the controls, thus providing an objective reference point for the analysis. This study was conducted exclusively amongst subjects residing in the same geographical latitude (42°N) thus ensuring uniformity of exposure to solar radiation. All subjects receiving vitamin D or calcium supplements were excluded from the baseline analysis; and were only employed in the determination of the effect of vitamin D supplementation on 25(OH)D concentration in IBS. Subjects’ anthropometric parameters were expressed as z-score and all analyses were adjusted for confounders.

The importance of this study was to initiate the first steps in the critical assessment of the role of vitamin D as an adjunctive therapy in children and adolescents with IBS. More studies are needed to determine whether adequate vitamin D supplementation can improve the quality of life indices in these children and adolescents by decreasing the rate of symptom flares and the occurrence of psychosomatic manifestations[2]. This hypothesis is supported by studies showing an association between psychiatric distress and hypovitaminosis D in youth[47], the improvement of symptoms in youth with depression and other psychiatric illnesses who received vitamin D supplementation[48–51], improvement in the quality of life of adult patients with IBS who received vitamin D supplementation[2, 33, 52], and improved depressive symptoms in youth with cystic fibrosis who received vitamin D supplementation[53].

## Conclusions

In this study, pediatric patients with IBS had significantly lower 25(OH)D concentration compared to controls despite having similar mean BMI values as controls. Only 7% of the children and adolescents with IBS were vitamin D sufficient, and >50% of the subjects with IBS had vitamin D deficiency. This is a much higher prevalence of vitamin D deficiency compared to IBD and other malabsorption syndromes. This high prevalence of vitamin D deficiency in IBS may due to a combination of factors such as restricted food choices, lifestyle habits that limit exposure to sunshine, female sex, and the possible role of hypoalbuminemia. A close monitoring of vitamin D status, and prompt repletion in cases of deficiency, should form part of routine clinical care of patients with IBS. Large randomized control trials are warranted to accurately determine the effect of optimal vitamin D supplementation on the intestinal and extra-intestinal manifestations of IBS.
