# Correction: Vitamin D status in pediatric irritable bowel syndrome

**DOI:** 10.1371/journal.pone.0173779

**Published:** 2017-03-09

**Authors:** Benjamin Udoka Nwosu, Louise Maranda, Ninfa Candela

The Data Availability statement for this paper is incorrect. The correct statement is: Our study data files are publicly deposited in the University of Massachusetts Medical School’s institutional repository, eScholarship@UMMS. Please access the data at either of the following links:

http://dx.doi.org/10.13028/M27P4Q

http://escholarship.umassmed.edu/pediatrics_data/4/
